# Double burden of malnutrition among women of reproductive age in Bangladesh: A comparative study of classical and Bayesian logistic regression approach

**DOI:** 10.1002/fsn3.3209

**Published:** 2023-01-06

**Authors:** Md. Ismail Hossain, Azizur Rahman, M. Sheikh Giash Uddin, Faozia Afia Zinia

**Affiliations:** ^1^ Department of Statistics Jagannath University Dhaka Bangladesh; ^2^ Department of Statistics Jahangirnagar University Savar, Dhaka Bangladesh

**Keywords:** Bayesian inference, classical inference, DBM, historical prior, malnutrition

## Abstract

Although the prevalence of undernutrition among women of reproductive age has declined in Bangladesh, the increase in the prevalence of overnutrition remains a major challenge. To achieve Sustainable Development Goal 2.2, it is important to identify the drivers of the double burden of malnutrition on women in Bangladesh. The Bangladesh Demographic and Health Survey, 2017–2018 was used to model the relationship between the double burden of malnutrition among women and the risk factors using a logistic regression model under the classical and Bayesian frameworks and performed the comparison between the regression models based on the narrowest confidence interval. Regarding the Bayesian application, the Metropolis‐Hastings algorithm with two types of prior information (historical and noninformative prior) was used to simulate parameter estimates from the posterior distributions. The Boruta algorithm was used to determine the significant predictors. Almost half of reproductive aged women experienced a form of malnutrition (12% were underweight, 26.1% were overweight, and 6.8% were obese). In terms of the narrowest interval estimate, it was found that Bayesian logistic regression with informative priors performs better than the noninformative priors and the classical logistic regression model. Women who were older, highly educated, from rich families, unemployed, and from urban residences were more likely to experience the double burden of malnutrition. This study recommended using the historical prior as the informative prior rather than the flat/noninformative prior to estimating the parameter uncertainty if historical data are available. The double burden of malnutrition among women is a major public health challenge in Bangladesh. This study was to determine the impact of effective risk factors on the double burden of malnutrition among women by applying the Bayesian framework. Using both informative and noninformative priors, “historical prior” was proposed as informative prior information. The main strength is that the proposed prior (historical prior) provided improved estimation as compared to the flat prior distribution.

## Graphical abstract

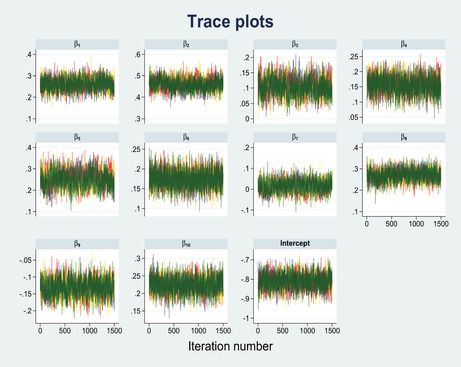

## 1 INTRODUCTION

Over the past few decades, several low‐ and middle‐income countries have managed to reduce women's undernutrition, but overnutrition (overweight and obesity) has become more prevalent (Hruby & Hu, 2015). Currently, many developed and underdeveloped countries are experiencing a coexistence of undernutrition and overnutrition known as the Double Burden of Malnutrition (DBM; Popkin et al., 2020). The double burden of malnutrition may occur at the individual level (an unnourished child can be overweight or obese when they reach adulthood), at the household level (coexistence of underweight children and overweight/obese adults in a household), and at the population level (presence of both undernutrition and overnutrition in the same community; World Health Organization, 2016). This study focused on the double burden of malnutrition on women at the population level.

Despite the low undernutrition rate, women's malnutrition is an emerging problem in most low‐ and middle‐income countries (LMICs) due to the high prevalence of overnutrition. Both undernutrition and overnutrition are associated with different types of health problems. For example, women suffering from overweight or obesity are affected by various noncommunicable diseases such as diabetes, hypertension, and cardiovascular diseases (Kominiarek & Peaceman, 2017). On the other hand, various pregnancy‐related difficulties are associated with undernourished health status (Nguyen, 2019). Globally, 39% of the population is overweight and 13% obese, and 31% of global deaths are from cardiovascular diseases (Oyekale, 2019; World Health Organization, 2021). Maternal nutrition is important for the optimal neurological development of the offspring (Peleg‐Raibstein, 2021). Maternal obesity was also associated with reduced cognitive scores in children (Pugh et al., 2015). Again, malnutrition and poor health are affecting the health of 462 million people in developing countries, and most of them are women and children (Amugsi et al., 2019; Pee et al., 2017). Many countries in sub‐Saharan Africa and southern Asia struggle with the double burden of malnutrition (Were et al., 2020). According to a previous estimate, in sub‐Saharan Africa, 18% of adults in African countries were underweight, while 15.5% were overweight or obese (Abarca‐Gómez et al., 2017). Malnutrition among women is recognized as a public health challenge in Ethiopia (Delbiso et al., 2016). The prevalence of overweight and obesity is increasing in Asia, including China, India, Pakistan, and Indonesia (Ng et al., 2014). Several other studies confirmed that the double burden of malnutrition is common among women in Bangladesh (Hasan et al., 2017). The prevalence of underweight decreased significantly between 2004 and 2014, while the prevalence of overweight and obesity increased during the same period (Tanwi et al., 2019).

Several studies attempted to uncover risk factors for the double burden of malnutrition among women. In general, poverty and education were seen as major drivers of the double burden of malnutrition among women of reproductive age (Delisle & Batal, 2016; Rahman et al., 2019). Previous research found that women's age, employment status, regional differences, and marital status were the important sociodemographic and economic factors that had already been identified as being associated with a woman being under or overweight in Bangladesh (Bishwajit, 2017; Rahman et al., 2019; Zahangir et al., 2017). In the past, many researchers in Bangladesh used classical inference to determine the risk factors associated with the double burden of malnutrition, where the unknown parameter was estimated using the maximum likelihood estimation procedure (Anik et al., 2019; Tanwi et al., 2019). However, Bayesian inference produces accurate estimates and captures more uncertainty than maximum likelihood estimation by introducing the prior information (Gebrie & Dessie, 2021). A Bayesian analysis combines prior information with data to produce posterior estimates and is built on Bayes' rules and theory. Although almost all researchers use flat/noninformative priors for Bayesian inference, which are essential functions of the data and almost give the same estimate as classical inference in most cases. On the other hand, using an informative prior distribution (also known as “historical prior information”) extracted from previous or historical data may improve the precision of the unknown parameter estimate (Hobbs et al., 2012). Statistical models using historical priors have been applied to other health science fields, such as analyzing microarray data (Li et al., 2015). To the best of our knowledge, Bayesian inference models with historical priors were not applied to examine the double burden of malnutrition among women of reproductive age in Bangladesh.

Researchers are increasingly recommending the use of Bayesian methods in social sciences and public health research to improve the interpretation of results (Lynch, 2011; Stern, 2016), and tools for Bayesian analysis have become increasingly accessible. For example, Bayesian modeling frameworks are available in SAS PROC MCMC, STATA, and R.

Based on the difference between classical inference and Bayesian inference, this study applied and compared both classical and Bayesian (using prior informative and prior non‐informative) statistical techniques to identify risk factors for the double burden of malnutrition among women of reproductive age in Bangladesh.

## 2 MATERIALS AND METHODS

### 2.1 Data source

This study used data from the Bangladesh Demographic and Health Survey (BDHS), 2017–2018, which was implemented by the National Institute of Population Research and Training (NIPORT) and funded by the United States Agency for International Development (USAID). This survey dataset is available at https://dhsprogram.com/data/available‐datasets.cfm.

### 2.2 Sample design

This cross‐sectional survey was conducted in urban and rural areas of Bangladesh and used a two‐stage stratified sampling design. In the first stage, 675 enumeration areas were selected, and in the second stage, 30 households were selected from each enumeration area. The survey was conducted in 20,250 households and 20,108 completed interviews with women 15–49 years of age. Women who were pregnant at the time of the survey were excluded from the analysis. Due to the collection of samples from a finite population, the estimation procedure and testing of the data needed suitable sampling weight adjustment. Data were weighted to represent the more accurate structure of the Bangladeshi population for further analysis purposes, using weighting factors provided by the Bangladesh Demographic and Health Survey. After weighting, 18,328 women of reproductive age were included in this study whose body mass index was measured (5170 from urban residences and 13,159 from rural residences in Bangladesh).

### 2.3 Dependent variable

The dependent variable for this study was “Double burden of malnutritional status among women of reproductive age”, which was assessed based on body mass index (BMI). BMI is defined by,

$$\mathrm{BMI}=\frac{\text{Weight in}\;\mathrm{kg}}{{\left(\text{High}t\;\text{in meter}\right)}^{2}}$$

According to the World Health Organization (WHO), this study categorized the body mass index value into four categories, such as,

$$\mathrm{BMI}=\left\{\begin{array}{c} \text{Underweight},\text{If}\;\mathrm{BMI}<18.5\;\mathrm{kg}/m^{2}\\ \text{Normal weight},\text{If}\;18.50\;\mathrm{kg}/m^{2}\leq \mathrm{BMI}\leq 24.90\;\mathrm{kg}/m^{2}\\ \begin{array}{c} \text{Overweight},\text{If}\;24.90\;\mathrm{kg}/m^{2}<\mathrm{BMI}\leq 29.90\;\mathrm{kg}/m^{2}\;\\ \text{Obese},\text{If}\;\mathrm{BMI}>29.90\;\mathrm{kg}/m^{2} \end{array} \end{array}\right.$$

Since the double burden of malnutrition indicates the presence of both undernutrition (underweight) and overnutrition (overweight, obese) at the same population, this study recodes the double burden of malnutrition (DBM) as,

$$\begin{array}{c} \mathrm{DBM}=\left\{\begin{array}{c} 1;\text{Coexistence of undernutrition and overnutrition within same population}\\ 0;\text{Otherwise}\; \end{array}\right. \end{array}$$

### 2.4 Explanatory variables

Multiple socio‐demographic and economic variables were included as independent/explanatory variables, such as women age in years, women education, employment status, marital status, mass media access, wealth status, religion, residence, and divisions.

### 2.5 Statistical analysis

#### 2.5.1 Boruta algorithm

This study considered the Boruta algorithm, first introduced by Miron Kursa and Witold Rudnicki, which was performed to extract the relevant risk factors for women's malnutrition from the set of explanatory variables. This is a wrapper‐built algorithm around the random forest classifier to find out the relevance and important variables with respect to the dependent variable. The importance measure of an attribute for all trees in the forest is obtained as the loss of accuracy of classification caused by the random permutation of attribute values between objects. Hereafter, the algorithm iteratively removes the variables which are proved by a statistical test to be less relevant than random probes (Kursa & Rudnicki, 2010).

#### 2.5.2 Univariate, bivariate, and multivariate analysis

A simple descriptive analysis, bivariate analysis, and multivariate analysis were conducted in this study. Descriptive analysis describes the percentage distribution of the variables. In bivariate analysis, this study examined the association between the double burden of malnutrition status among reproductive aged women and important independent variables that were selected by the Boruta algorithm. In this case, the chi‐square test statistic is applied, and it can be defined as,

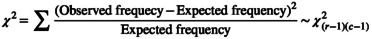

where *r* is the number of categories for the independent variable and *c* is the number of categories for the dependent variable.

In a multivariate setup, the effect of an independent variable on the double burden of malnutrition status among women aged between 15 and 49 was determined using logistic regression. This study applied classical logistic regression as well as Bayesian logistic regression to identify the risk factors for double burden of malnutrition among women of reproductive age in Bangladesh.

#### 2.5.3 Classical logistic regression

Let $D_{i}$ denote the binary dependent variable for the *i*th observation, and $E_{i1},\ldots ,E_{\mathrm{ip}}$ be a set of explanatory variables which can be quantitative or indicator variables referring to the level of categorical variables. Since $D_{i}$ is a binary variable, it has a Bernoulli distribution with parameter $\pi_{i}$. The dependent of the probability of success on independent variables is assumed to be respectively as,

$$P\left(D=1\right)=\pi_{i}=\frac{\exp\left(\beta_{0}+\beta_{1}E_{i1}+\ldots +\beta_{p}E_{\mathrm{ip}}\right)}{1+\exp\left(\beta_{0}+\beta_{1}E_{i1}+\ldots +\beta_{p}E_{\mathrm{ip}}\right)}$$

The above relation also can be expressed as,

$$\text{logit}\left(\pi_{i}\right)=\log\frac{\pi_{i}}{1-\pi_{i}}=\beta_{0}+\beta_{1}E_{i1}+\ldots +\beta_{p}E_{\mathrm{ip}}$$

The odds ratio with a 95% confidence interval was usually used to explain predictor variables impact.

#### 2.5.4 Bayesian logistic regression

Bayesian logistic regression, an alternative to the classical logistic regression analysis, is conducted based on Bayes theorem which can be defined as,

$$f\left(\beta |D_{i},E_{\mathrm{ip}}\right)=f\left(D_{i}|\beta \right)\times f\left(\beta \right)$$

Here, $f\left(\beta |D_{i},E_{\mathrm{ip}}\right)$ be the posterior distribution of the parameters β, $f\left(D_{i}|\beta \right)$ be the likelihood function, and $f\left(\beta \right)$ be the prior distribution of parameter β.

Since, $D_{i}$ be the binary dependent variable, and $E_{i1},\ldots ,E_{\mathrm{ip}}$ be a set of explanatory variables, and it has a Bernoulli distribution with parameter $\pi_{i}$. So, the link function can be written as,

$$\text{logit}\left(\pi_{i}\right)=\log\frac{\pi_{i}}{1-\pi_{i}}=\beta_{0}+\beta_{1}E_{i1}+\ldots +\beta_{p}E_{\mathrm{ip}}$$

where

$$\pi_{i}=\frac{\exp\left(\beta_{0}+\beta_{1}E_{i1}+\ldots +\beta_{p}E_{\mathrm{ip}}\right)}{1+\exp\left(\beta_{0}+\beta_{1}E_{i1}+\ldots +\beta_{p}E_{\mathrm{ip}}\right)}$$

Using the value of $\pi_{i}$, the likelihood function $f\left(D_{i}|\beta \right)$ can be written as,

$$f\left(D_{i}|\beta \right)=\prod \left(\begin{array}{c} M_{i}\\ D_{i} \end{array}\right){\left(\frac{\exp\left(\beta_{0}+\beta_{1}E_{i1}+\ldots +\beta_{p}E_{\mathrm{ip}}\right)}{1+\exp\left(\beta_{0}+\beta_{1}E_{i1}+\ldots +\beta_{p}E_{\mathrm{ip}}\right)}\right)}^{D_{i}}{\left(1-\frac{\exp\left(\beta_{0}+\beta_{1}E_{i1}+\ldots +\beta_{p}E_{\mathrm{ip}}\right)}{1+\exp\left(\beta_{0}+\beta_{1}E_{i1}+\ldots +\beta_{p}E_{\mathrm{ip}}\right)}\right)}^{M_{i}-D_{i}}$$

The Bayesian analysis combines the information in the data represented by the entire likelihood function with prior knowledge about the unknown parameters, which may come from other data sets or a modeler's experience and physical intuition. This study used two types of prior information, (a) flat/noninformative prior, (b) informative prior (which was obtained from previous BDHS survey data).

This study used the most common priors for logistic regression parameters, which are of the form

$$\beta_{j}\sim N\left(\mu_{j},\sigma_{j}^{2}\right)$$

In terms of noninformative specification, the most common choice is $\mu_{j}=0$, and $\sigma_{j}^{2}=10^{6}$ (large enough; Gebrie, 2021). In the case of informative prior specification, this study applies maximum likelihood estimation procedure to estimate the unknown parameter from previous survey dataset (i.e., Bangladesh Demographic and Health Survey, 2014, https://dhsprogram.com/data/available‐datasets.cfm). Then the parametric bootstrap, a popular resampling technique to estimate summary statistics (mean or standard deviation) on a population by sampling a dataset with replacement, was used for the efficient computation of Bayes prior distributions (Efron, 2012).

In the case of Bayesian MCMC (Markov Chain Monte Carlo) approximation, this study used the Metropolis‐Hastings algorithm to estimate the marginal posterior distribution for unknown parameters. The expected value of the posterior distribution of parameters $\beta_{j}$ will be considered as regression coefficients of the Bayesian logistic model, and it can provide credible intervals for parameters that are more easily interpreted than the concept of confidence interval in classical inference. Note that the parameter estimates are subject to Monte Carlo error, which is difficult to quantify. Therefore, this study has chosen a very long run of which convergence was reached at 150,000 (per chain) after a burn‐in period of 500 and thinning of every 99th element of the chain for each model. In MCMC sampling, values are drawn from a probability distribution. The distribution of the current value drawn depends on the previously drawn value (but not on values before that). Once the chain has converged, its elements can be seen as a sample from the target posterior distribution. To evaluate the convergence of MCMC chains it is helpful to create multiple chains that have different starting values. In this study, the total number of Markov chains was 4. This study also used trace plots for checking and interpreting the results of the convergency of MCMC sampling.

### 2.6 Model comparison

One of the objectives of this study was to compare three logistic regression models (classical, Bayesian with noninformative priors, and Bayesian with informative priors) and find the best one among these three models on the basis of the interval estimation criterion. A good model will have a relatively narrow confidence interval. A narrow confidence interval implies that there is a smaller chance of obtaining an observation within that interval, therefore, the model accuracy is higher.

### 2.7 Analytical software

Data wrangling, descriptive analysis, and bivariate analysis were performed in SPSS (version 25), and model fitting (both classical and Bayesian) was performed in STATA 16. This study used the STATA package “bayesmh” for Bayesian logistic regression. The Boruta algorithm was implemented to select risk factors using the Boruta package in the R‐programming (version 4.0) language.

## 3 RESULTS

### 3.1 Percentage of four categories of women's nutritional status

**FIGURE 1:**
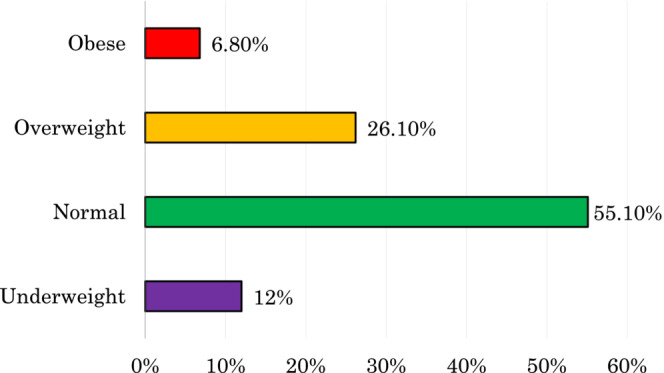
Nutrition status among women of reproductive age in Bangladesh.

Based on the World Health Organization (WHO) criteria, this study categorized the body mass index value into four categories, such as underweight, normal, overweight, and obese. Figure 1 shows that the prevalence of underweight women was 12%, normal‐weight women was 55.1%, overweight women was 26.1%, and obese women was 6.8%. The prevalence of a double burden of women's malnutrition (underweight as well as overweight and obese) was approximately 45%.

### 3.2 Percentage distribution of selected variables

Table 1 shows the percentage distribution of women according to the selected socio‐economic and demographic characteristics. Slightly less than one‐third (28.2%) of women lived in rural areas, while over two‐thirds (71.8%) were in urban areas of Bangladesh. Most of the women reside in the Dhaka division (25.2%) rather than the Chattogram division (17.7%). Results from Table 1 show that percentage of women aged 35–49 years was higher (39.8%), and most of the women were married (93.9%). With regard to education level, 38.8% of women had achieved a secondary level of education and only 11.9% were at a higher education level. The proportion of employed and unemployed women was equal (approximately 50%). In terms of household wealth status, a larger percentage of women are in rich households (41.4%). Among the total sample, the percentage of Muslims was 90.5%, and the proportion of women with exposure to mass media was 55.8%.

**TABLE 1:** Sociodemographic and demographic characteristics of the study participants.

Variables	Frequency (*n* = 18,328)	Percentage
Women age (in years)		
15–24	4626	25.2
25–34	6399	34.9
35–49	7303	39.8
Women education		
No education	3220	17.6
Primary education	5815	31.7
Secondary education	7116	38.8
Higher	2178	11.9
Employment status		
Employed	9035	49.3
Unemployed	9293	50.7
Marital status		
Married	17,212	93.9
Others	1117	6.1
Wealth status		
Poor	7033	38.4
Middle	3705	20.2
Rich	7590	41.4
Religion		
Islam	16,581	90.5
Others	1747	9.5
Division		
Barisal	1024	5.6
Chattogram	3248	17.7
Dhaka	4611	25.2
Khulna	2163	11.8
Mymensingh	1401	7.6
Rajshahi	2607	14.2
Rangpur	2211	12.1
Sylhet	1063	5.8
Mass media access		
Exposed	10,219	55.8
Not exposed	8109	44.2
Residence		
Rural	13,159	71.8
Urban	5170	28.2

### 3.3 Variables selection

**FIGURE 2:**
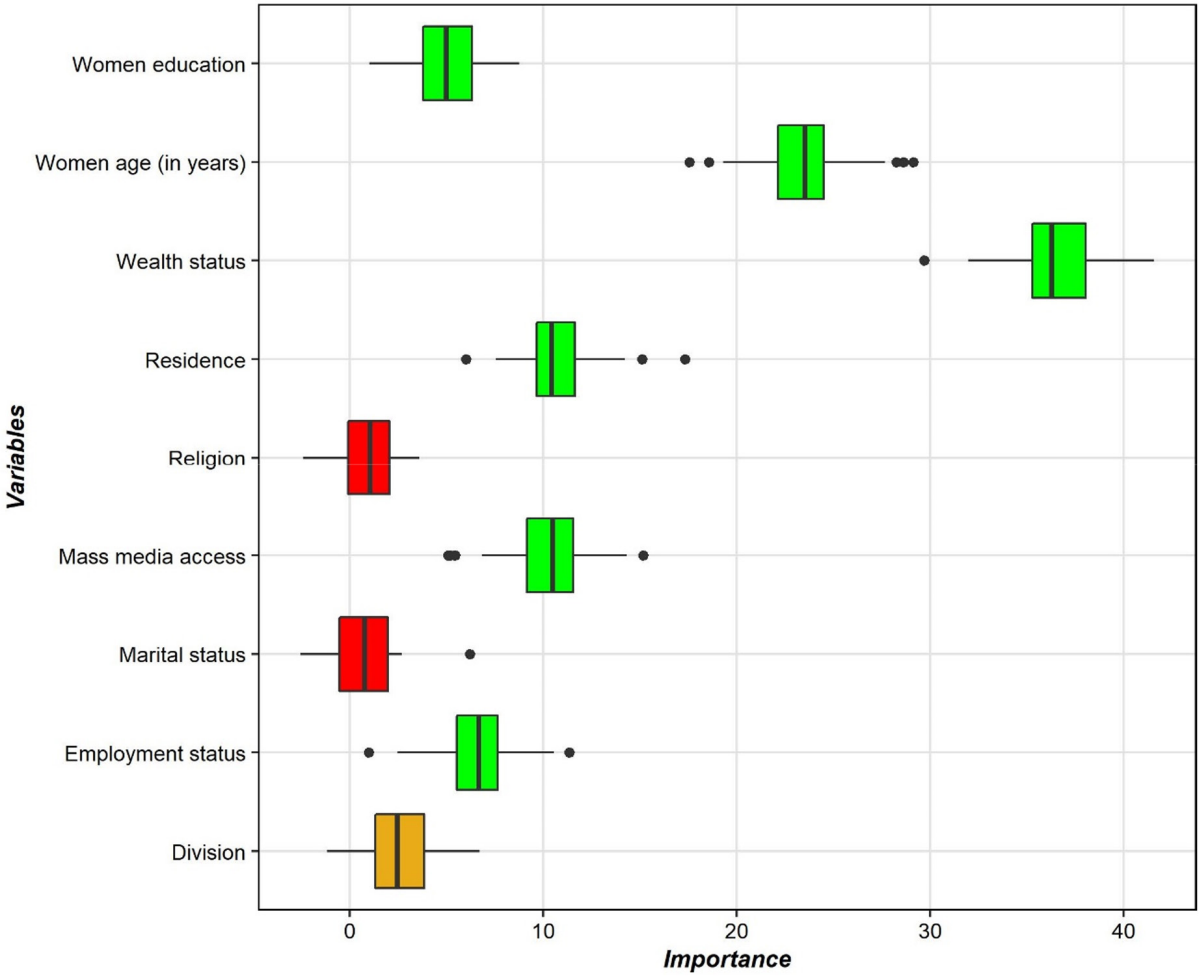
Variable selection using the Boruta algorithm.

Figure 2 shows that using the Boruta algorithm, six variables (women age (in years), women education, employment status, wealth status, mass media access, and residence) were selected as the most important (green box plot) variables from nine variables as risk factors. This study does not contain any tentative characteristics (yellow box plot) or unimportant characteristics (red box plot).

### 3.4 Bivariate analysis of selected variables on double burden of malnutrition

The prevalence of the double burden of malnutrition among 15–49 aged women and important characteristics (evaluated from the Boruta algorithm) is manifested in Table 2. In accordance with the $\chi^{2}$ test, all factors were significantly associated (*p* < .001) with the double burden of malnutrition among women of reproductive age. The prevalence of double burden of malnutrition among women was found to be higher in urban residences (52.5%), women aged 35–49 years (49.3%), rich households (52.7%), women with a higher educational level (50%), unemployed women (48%), and women who had access to the media (48.7%).

**TABLE 2:** Percentage distribution and association between selected variables and the double burden of malnutrition among women of reproductive age in Bangladesh.

Variables	Under nutrition (12%)	Over nutrition (32.9%)	Double burden of women's malnutrition			
			Yes (44.9%)	No (55.1%)	$\chi^{2}$‐value	*p*‐Value
Women age (in years)						
15–24	18.4	18.5	36.9	63.1	179.50	<.001
25–34	9.6	36.1	45.7	54.3		
35–49	10.0	39.3	49.3	50.7		
Women education						
No education	15.2	25.6	40.7	59.3	45.97	<.001
Primary education	13.1	31.7	44.8	55.2		
Secondary education	10.8	34.5	45.3	54.7		
Higher	7.9	42.1	50.0	50.0		
Employment status						
Employed	13.1	28.7	41.7	58.3	73.21	<.001
Unemployed	10.9	37.1	48.0	52.0		
Wealth status						
Poor	18.3	19.8	38.1	61.9	3331.73	<.001
Middle	10.6	31.2	41.8	58.2		
Rich	6.7	46.0	52.7	47.3		
Mass media access						
Exposed	8.6	40.0	48.7	51.3	131.78	<.001
Not exposed	16.1	24.0	40.2	59.8		
Residence						
Rural	13.3	28.7	41.9	58.1	166.50	<.001
Urban	8.6	43.8	52.5	47.5		

Table 2 also presents the prevalence of under‐nutrition and over‐nutrition (overweight and obese) according to the background characteristics. The prevalence of undernutrition was found to be higher for women aged between 15 and 24 years (18.4%), whereas women aged 35–49 years showed a higher prevalence of overnutrition (approximately 39.3%). Also, uneducated women suffered more from undernutrition (15.2%), whereas the prevalence of overnutrition was higher for highly educated women in Bangladesh (42.1%). Since employed women were engaged in more physical activity, and as a result, the prevalence of undernutrition was higher among employed women (13.1%). On the other hand, less physical activity increases the body weight, and from Table 2 it can be noted that the percentage of over‐nourished women was found to be higher for unemployed women (37.1%). In accordance with wealth status, women from rich households had a lower percentage of undernutrition (approximately 7%) but a higher percentage of overnutrition (46%). Women who were not accessed by any type of media like TV, radio, or newspapers had a 16.1% undernutrition prevalence compared to others. Table 2 revealed that the prevalence of undernutrition in rural residence was higher (approximately 13%), and overnutrition in urban areas was approximately 15% higher than in rural residence of Bangladesh.

### 3.5 Identifying factors contributing to double burden of malnutrition

This study tried to fit three binary logistic regression models (Model 1, Model 2, and Model 3). Here, Model 1 was a classical binary logistic regression model, Model 2 and 3 was a Bayesian binary logistic regression model. According to Bayes theorem, in Bayesian analysis there was a need for a prior distribution. This study used both noninformative/flat prior (Model 2) and informative/historical prior (Model 3). Historical prior was generated by using Bangladesh Demographic and Health Survey, 2014 survey data set. After fitting these three models, they were compared in terms of interval estimate of the unknown parameters. Short confidence interval increases the accuracy of the model. Based on this criterion, it was observed that Model 3 (i.e., Bayesian binary logistic regression with informative prior) was the best model for determining the factors associated with the double burden of malnutrition among reproductive aged women in Bangladesh. Table 3 represents the odds ratios with 95% confidence interval (credible interval) for both classical and Bayesian binary logistic regression analysis. Parameter estimation for Model 3 was carried out using the Markov Chain Monte Carlo (MCMC) via Metropolis‐Hastings Algorithm, where the number of Markov chain was 4. Convergence of the MCMC was reached at 150,000 iterations (per chain) after a burn‐in period of 500 samples and thinning of every 99th element of the chain, and it was traced through the trace plot.

**TABLE 3:** Odds ratios (OR) and 95% confidence interval (95% CI) from classical logistic regression and Bayesian (noninformative and informative prior) logistic regression.

Variables	Model 1	Model 2	Model 3
	OR (95% CI)	OR (95% CI)	OR (95% CI)
Women age (in years)			
15–24 (Ref.)	1	1	1
25–34	1.52 (1.40, 1.65)	1.51 (1.39, 1.64)	1.30 (1.23, 1.38)
35–49	1.92 (1.76, 2.09)	1.91 (1.75, 2.08)	1.59 (1.50, 1.68)
Women education			
No education (Ref.)	1	1	1
Primary education	1.23 (1.12, 1.34)	1.22 (1.11, 1.34)	1.11 (1.05, 1.18)
Secondary education	1.29 (1.17, 1.42)	1.29 (1.17, 1.43)	1.17 (1.10, 1.24)
Higher	1.42 (1.25, 1.60)	1.39 (1.21, 1.61)	1.29 (1.19, 1.40)
Employment status			
Employed (Ref.)	1	1	1
Unemployed	1.20 (1.13, 1.28)	1.19 (1.12, 1.27)	1.19 (1.14, 1.24)
Wealth status			
Poor (Ref.)	1	1	1
Middle	1.06 (0.97, 1.16)	1.06 (0.97, 1.16)	1.02 (0.96, 1.08)
Rich	1.41 (1.30, 1.54)	1.44 (1.30, 1.57)	1.31 (1.24, 1.39)
Mass media access			
Exposed (Ref.)	1	1	1
Not exposed	0.87 (0.82, 0.94)	0.88 (0.82, 0.94)	0.88 (0.83, 0.92)
Residence			
Rural (Ref.)	1	1	1
Urban	1.20 (1.12, 1.28)	1.20 (1.12, 1.29)	1.25 (1.20, 1.31)

*Note*: Model 1 = Classical binary logistic regression. Model 2 = Bayesian binary logistic regression with noninformative prior. Model 3 = Bayesian binary logistic regression with informative prior.

From the results of Bayesian inference (Model 3), this study identified that the respondent current age was positively and significantly associated with the double burden of women's malnutrition. Women with age group 25–34 years have 30% higher odds of double burden of malnutrition than younger aged women (i.e., 15–24 years). The odds ratio was greater for 35–49 years aged women (OR = 1.59, 95% CI: 1.50, 1.68).

Compared with uneducated women, women who had formal/primary education, secondary education, and higher education were 11% (OR = 1.11 for primary education), 17% (OR = 1.17 for secondary education), and 29% (OR = 1.29 for higher education) more risk to have double burden of malnutrition.

In terms of respondent working status, it has a positive impact on the double burden of malnutrition among reproductive aged women in Bangladesh. That is, unemployed women have 19% higher risk (OR = 1.19, 95% CI: 1.14, 1.24) of double burden of malnutrition than the employed women.

It was observed that women who belonged to middle class and rich families had 1.02 and 1.31 times more likely to be double burden of malnourished than women who belonged in poor families. That is, there was a significant and positive impact between household wealth status and double burden of malnutrition among women of reproductive age in Bangladesh.

Using the odds ratio of Model 3, this study found mass media has negative impact on double burden of malnutrition among women. According to the results, it can be stated that, women who were not accessed by media had 12% lower chance (OR = 0.88, 95% CI: 0.83, 0.92) of double burden of malnutrition (i.e., undernutrition as well as overnutrition) than women who were attached by media.

From the estimate of Bayesian logistic regression with informative prior, this study found the place of residence has a positive impact on the double burden of malnutrition among women, which means that the respondents living in the urban areas have significantly 25% higher risk (OR = 1.25, 95% CI: 1.20, 1.31) of double burden of malnutrition than respondents who live in rural areas of Bangladesh.

**FIGURE 3:**
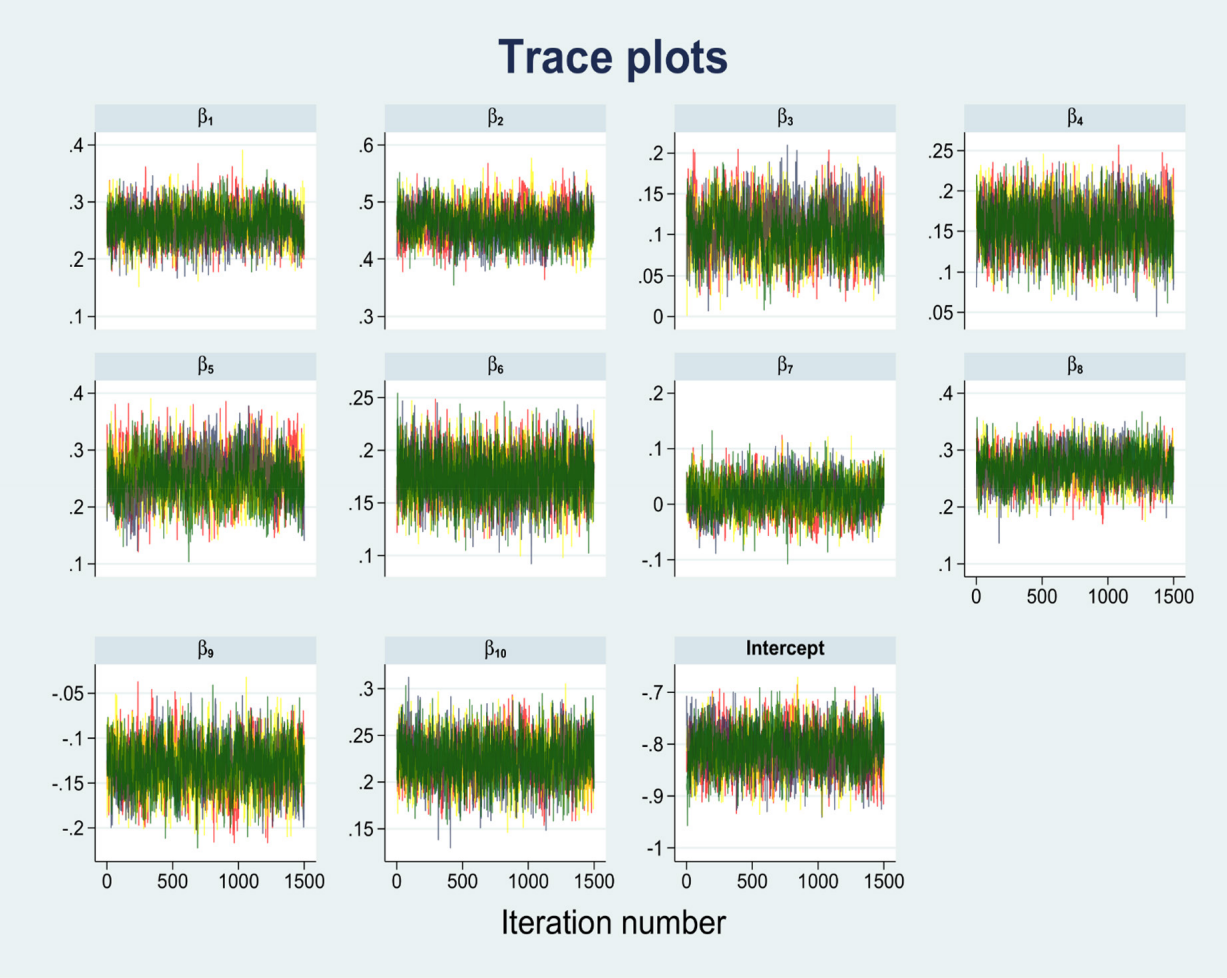
Trace plots for model parameters of the Bayesian logistic regression model with informative prior (historical prior).

Trace plots of the samples are very important to check the evaluation of the convergence of the MCMC sampling algorithm, and the fluctuation of the trace plots shows the balance of the numerical distribution of the parameter. Figure 3 shows the posterior distribution for the coefficients of the model parameters under the informative prior distributions. The MCMC trace plots of model parameters are illustrating how well the samples are overlapped. This indicates that the Bayesian inference was successful as well as the informative prior distributions used are accurate, and the Markov chain has converged. The Gelman‐Rubin diagnostic evaluates MCMC convergence by analyzing the difference between multiple Markov chains. The convergence is assessed by comparing the estimated between‐chains and within‐chain variances for each model parameter. According to Brooks and Gelman (1998), if the Gelman‐Rubin diagnostic statistics ($R_{c}$) is less than 1.1 for all model parameters (β), one can be fairly confident that convergence has been reached. In this study, the value of $R_{c}$ was less than 1.1, for all $\beta_{i};i=1,\ldots ,10$. Here, *β*_1_ = 25–34 years women, *β*_2_ = 35–49 years women, $\beta_{3}=\text{Primary education}$, $\beta_{4}=\text{Secondary education}$, $\beta_{5}=\text{Higher education}$, $\beta_{6}=\text{Unemployed women}$, $\beta_{7}=\text{Middle wealth status}$, $\beta_{8}=\text{Rich wealth status}$, $\beta_{9}=\text{Mass media not access}$, and $\beta_{10}=\text{Urban residence}$.

## 4 DISCUSSION

Although Bangladesh has made progress in reducing undernutrition among women of reproductive age, the rapid increase in overnutrition is a major public health challenge. Our study investigated the prevalence and risk factors of the double burden of malnutrition (i.e., the coexistence of undernutrition and overnutrition) among women of reproductive age in Bangladesh by applying both classical and Bayesian inference models with informative (historic) and noninformative (flat) priors. The Bayesian logistic regression model with informative priors proved to have the narrowest interval estimate, which suggests the model had superior precision and was less biased (Tekin et al., 2018). The historical prior/informative prior derived from the 2014 Bangladesh Demographic and Health Survey improves the model's performance.

According to the results of this study, the prevalence of the double burden of malnutrition among women of reproductive age in Bangladesh was approximately 45%. This percentage was higher than Ghana (Kushitor et al., 2020). Earlier studies have reported that the prevalence of the double burden of household‐level malnutrition was high in LMICs (Doku & Neupane, 2015; Onyango et al., 2019). We estimate the prevalence of undernutrition to be approximately 12% women in Bangladesh being underweight, which was consistent with another recent study conducted in Bangladesh (Rahman et al., 2022). Compared to undernutrition, approximately 33% women experienced overnutrition (i.e., overweight and obese), which was higher than several South Asian countries (He et al., 2016; Hong et al., 2018).

Using Bayesian inference with informative prior findings, this study found a significant, positive, and increasing association between age and double burden of malnutrition among women. Women older than 24 years of age were at higher risk of double burden of malnutrition. That is, older women were more likely to experience double burden of malnutrition. This finding was supported by previous studies conducted in Japan and Ghana (Doku & Neupane, 2015; Negoro et al., 2015).

There was a significant positive interconnection between education and the double burden of malnutrition among women of reproductive age in Bangladesh. According to the prevalence analysis of this study, it can be highlighted that women with higher education have a lower prevalence of undernutrition and a higher prevalence of overnutrition. Since double burden of malnutrition is coexistence of undernutrition and overnutrition at the same population, the actual prevalence and chances of double burden of malnutrition were higher at highly educated women in Bangladesh. Consistent with earlier study by Doku and Neupane (2015), it appears that highly educated women may have more knowledge about how to overcome undernutrition. On the other hand, because of their higher income and greater independence, they may lead lives with less physical activity and better access to hypercaloric foods, which are considered to be the cause of overweight as well as obesity.

According to this study, an unemployed woman was more likely to suffer from a double burden of malnutrition than an employed woman because of the high prevalence of overnutrition. A similar result was found in an Ethiopian study conducted in 2016 and revealed that less physical activity and the intake of energy‐dense foods may also increase body weight (Abrha et al., 2016). Another study explained that increasing women's employment has great potential to improve women's nutritional status (Gillespie & Bold, 2017). An interesting finding of this study was that women who did not have access to media were less likely to suffer from the double burden of malnutrition. Although Fox et al. (2018) found the media campaigns improve the knowledge and nutritional health behavior, other research studies shown media use was associated with increased sedentary behavior and decreased physical activity (Jordan et al., 2008; Matusitz & McCormick, 2012).

The double burden among women of reproductive age was positively associated with the household wealth index. There was a lower rate of undernutrition for women with a rich wealth index. Wealthier families have better consistent access to food. On the other hand, the prevalence of overnutrition was so high that the prevalence of double burden of malnutrition was found to be higher for women from rich households. That is, women in the richest wealth index had the highest risk of suffering double the burden of malnutrition. This result was consistent with previous studies (Bishwajit, 2017; Biswas et al., 2017). Because of the increase in per capita income, middle and upper‐middle families may have adopted Western diets, with higher caloric foods. As a result, middle‐ and upper‐class families were at a higher risk of being overweight or obese, possibly explaining the double burden of malnutrition (Kishawi et al., 2016; Leroy et al., 2014).

This study also found that women living in urban areas were more likely to suffer a double burden of malnutrition than women living in rural areas. A recent comparative study between Bangladesh, Nepal, Pakistan, and Myanmar showed that the double burden of household‐level malnutrition was higher in urban than in rural areas (Anik et al., 2019).

## 5 STRENGTHS AND LIMITATIONS OF THE STUDY

This study applied Bayesian estimation with informative priors to examine the determinants of double burden of malnutrition among women of reproductive age in Bangladesh. A resampling technique, “bootstrapping” was used to estimate a precise/accurate uncertainty of the mean and variance of the prior distribution from historical data. The proposed prior (historical prior) provided improved estimate as compared to flat prior distribution. The study has used nationally representative survey data which has been collected using a comprehensive methodology under strict technical and ethical considerations which have increased the validity of the results. Despite these strengths, this study had some limitations. Firstly, this study applies only one technique to discover the informative prior distribution. Obviously, a prior distribution from various techniques with validation measure adds to the strength of this study. Secondly, due to data limitations, this study is unable to utilize several important factors that are major contributors to women malnutrition. Thirdly, the cross‐sectional characteristics of the data allow conclusions to be drawn about associations, but prevent them from being established causal links.

## 6 CONCLUSION

The double burden of malnutrition is now considered a major public health challenge due to the tremendous increase in overnutrition among reproductive‐aged women in Bangladesh. This study addresses this challenge and examines the risk factors associated with the double burden of malnutrition among women of reproductive age in Bangladesh. The Boruta algorithm was used to explain the importance of effective factors. Women's age, educational attainment, wealth status, employment status, exposure to mass media, and place of residence had the most significant impact on the double burden of malnutrition. Women from rural areas and the wealthiest households were found to be at a higher risk of having a double burden of malnutrition. So, to reduce the double burden of malnutrition, strategies need to be implemented, especially in residential as well as socio‐economic status contexts, and awareness‐raising programs about healthy living should be executed. In addition, this study compared classical and Bayesian frameworks when examining the double burden of malnutrition. Compared to the Bayesian framework, attempting to estimate the model parameters using classical techniques leads to estimation problems, inaccurate parameter estimates, and limitations in drawing conclusions. Using Bayesian inference with prior information offers advantages for quantifying uncertainties. In this study, we used two types of prior: a flat/noninformative prior and an informative prior. The historical prior derived from the results of the previous BDHS survey was used as an informative prior. The findings of our study indicated that the posterior distributions were generally stable across different prior distributions, but we found that using the historical prior was more appropriate than a flat/noninformative prior. Therefore, this study recommends using the historical prior distribution as prior information to increase the accuracy of the study results, and policymakers should pay attention to continuing this study in the future.

## FUNDING INFORMATION

This research did not receive any specific grant from funding agencies in the public, commercial, or not‐for‐profit sectors.

## CONFLICT OF INTEREST

The authors declare that they have no conflicts of interest.

## Data Availability

This study used data from Bangladesh Demographic and Health Survey (BDHS), 2017–2018, which is available from https://dhsprogram.com/data/available‐datasets.cfm.
